# Artificial Intelligence for Perioperative Risk Prediction in Anesthesiology: A Bibliometric and Knowledge-Mapping Analysis

**DOI:** 10.3390/healthcare14172785

**Published:** 2026-09-01

**Authors:** Mehmet Özkılıç

**Affiliations:** Department of Anesthesiology and Reanimation, Diyarbakır Gazi Yaşargil Training and Research Hospital, University of Health Sciences, 21070 Diyarbakır, Turkey; mehmet.ozkilic@saglik.gov.tr

**Keywords:** artificial intelligence, perioperative risk prediction, machine learning, anesthesiology, bibliometric analysis, science mapping, clinical decision support, explainable artificial intelligence

## Abstract

**Background:** Artificial intelligence (AI) is increasingly being investigated in perioperative medicine for risk stratification, complication prediction, patient safety, and clinical decision support. This study characterized the research landscape, intellectual structure, and thematic evolution of AI-based perioperative risk prediction in anesthesiology. **Methods:** Publications indexed in the Web of Science Core Collection between 2018 and 3 June 2026 were analyzed using Bibliometrix and Biblioshiny. Scientific productivity, citations, collaboration networks, keyword co-occurrence, thematic evolution, and Reference Publication Year Spectroscopy were evaluated. Partial 2026 data were excluded from the compound annual growth-rate calculation. **Results:** A total of 152 publications were included. Scientific production increased substantially, with a compound annual growth rate of 77.72% between 2018 and 2025. The United States and China led publication output, while the United States had the highest total citation count. Citation and thematic analyses identified perioperative outcome prediction, predictive hemodynamic monitoring, and explainable AI as important components of the field’s intellectual structure. Research attention increasingly shifted from methodological machine learning development toward clinically oriented topics, including mortality, postoperative complications, delirium, postoperative nausea and vomiting, and perioperative risk stratification. Emerging themes included explainable AI, large language models, natural language processing, and clinical decision-support systems. **Conclusions:** Research attention has increasingly shifted from exploratory model development toward clinically oriented risk-stratification and decision-support applications. Emerging directions emphasize interpretability, multimodal data integration, and individualized risk assessment. These bibliometric patterns reflect changes in research emphasis but do not demonstrate clinical adoption or effectiveness.

## 1. Introduction

One of the primary objectives of perioperative medicine is to accurately identify high-risk patients before complications occur and to guide clinical decision-making according to individualized risk profiles [1,2]. Nevertheless, adverse outcomes such as postoperative pulmonary complications, acute kidney injury, intensive care unit admission, prolonged hospitalization, and mortality continue to impose a substantial clinical and economic burden worldwide [3,4]. Although traditional scoring systems and regression-based models commonly used in perioperative risk assessment provide valuable information, they may be insufficient to fully capture the high-dimensional and nonlinear relationships among patient characteristics, comorbidities, laboratory findings, surgical factors, and intraoperative physiological variables [5,6]. The widespread availability of large-scale data derived from electronic health records and high-frequency monitoring systems has increased the demand for novel approaches capable of modeling these complex relationships, refining individualized risk stratification, and potentially informing targeted optimization strategies [7,8]. Beyond perioperative settings, these data-driven approaches may also extend to primary healthcare, where earlier risk identification could support preventive management before advanced or surgical care is required [5,6,7,8].

Within perioperative medicine, this growing need for data-driven risk assessment has led to the rapid adoption of artificial intelligence (AI), machine learning (ML), and deep learning (DL) techniques [1,9]. In recent years, AI-based models have demonstrated promising performance in predicting post-induction hypotension, acute kidney injury, postoperative pulmonary complications, intensive care mortality, and other adverse perioperative outcomes [5,10]. By learning from large-scale clinical datasets, these algorithms can model complex interactions among numerous variables and serve as valuable complements to traditional statistical approaches [6]. Collectively, these developments reflect a clear shift toward data-driven prediction systems in perioperative risk assessment.

Studies published between 2018 and 2020 represented a major turning point in the development of AI-based risk prediction research in anesthesiology [4,11]. Fritz et al. and Hill et al. demonstrated the feasibility of applying advanced analytical methods to postoperative mortality prediction [4,8], while Kendale et al. showed the potential of supervised machine learning for predicting post-induction hypotension [12].

The field has accelerated considerably in recent years. Particularly since 2023, the emergence of technologies such as explainable artificial intelligence, large language models, and clinical decision-support systems has shifted the research focus from algorithm development toward clinical integration [13,14,15]. Contemporary studies increasingly aim not only to improve predictive accuracy but also to enhance model transparency, clinician trust, and real-world applicability [7,16]. This trend suggests that AI-based perioperative risk prediction research has entered a new phase of growth and maturation.

This transformation in scientific output underscores the need for a systematic evaluation of the field’s research landscape [17]. Bibliometric analyses facilitate the assessment of a research field’s intellectual structure by identifying publication trends, citation relationships, research collaborations, influential researchers, institutions, and countries [18,19]. They also contribute to the identification of conceptual structures, thematic clusters, and emerging research areas [20,21]. In rapidly expanding and interdisciplinary fields, bibliometric methods are increasingly recognized as valuable tools for mapping existing knowledge ecosystems and identifying future research priorities [18,19,22].

Recent bibliometric studies have mapped AI research in perioperative medicine and anesthesiology from broader perspectives [18,19,22,23,24]. Chan et al. analyzed Scopus-indexed perioperative AI literature through October 2024 using VOSviewer, Biblioshiny, and Microsoft Excel, focusing on publication trends, collaborations, and research hotspots [18]. Xie et al. examined Web of Science Core Collection records through April 2024 using Excel, CiteSpace, and VOSviewer, covering diverse anesthesiology applications such as regional anesthesia, airway management, postoperative pain, depth-of-anesthesia monitoring, drug delivery, and predictive modeling [24]. Ou et al. analyzed Web of Science Core Collection publications from 2000 to 2023 using Excel and VOSviewer, while Liu et al. examined the same broad field from 2004 to 2024 using VOSviewer and CiteSpace, including productivity, collaboration, co-citation, and keyword-based analyses [19,22]. Complementary scoping and methodological studies have addressed specific applications, including transfusion prediction, perioperative anesthetic management in cardiac surgery, and natural language processing of preanesthetic records [25,26,27]. In contrast, the present study focuses specifically on AI-based perioperative risk prediction in anesthesiology and integrates productivity, citation, collaboration, thematic mapping, thematic evolution, and RPYS analyses within a focused 2018–2026 dataset.

To address this gap, the present study employed bibliometric and science-mapping approaches to analyze publications indexed in the Web of Science (WoS) that focused on AI-based risk prediction in anesthesiology. Scientific productivity trends, citation structures, research collaborations, intellectual foundations, conceptual structures, and thematic development were evaluated simultaneously. By doing so, this study aims to characterize the evolution of AI-based perioperative risk prediction research, identify influential contributors and emerging research areas, and provide a strategic perspective for future research and clinical applications.

## 2. Methods

### 2.1. Data Source and Search Strategy

The Web of Science Core Collection was used as the data source for this bibliometric analysis because it provides standardized bibliographic records and comprehensive citation data suitable for bibliometric and science-mapping studies.

The literature search was conducted on 3 June 2026, using the Advanced Search interface and the Topic field. The following search query was applied:

(“artificial intelligence” OR “machine learning” OR “deep learning”) AND (“risk prediction” OR “risk assessment” OR “risk stratification”) AND (“anesthesia” OR “anaesthesia” OR “anesthesiology” OR “anaesthesiology”)

The initial search identified 170 records. The results were sequentially restricted to publications indexed in the Science Citation Index Expanded (SCI-EXPANDED) or Emerging Sources Citation Index (ESCI), published between 1 January 2018 and the search date of 3 June 2026, classified as Articles or Review Articles, and written in English. No additional Web of Science category or subject-area restriction was applied. Following the application of these database-level restrictions, 152 records remained for thematic eligibility screening.

Eligibility assessment, data cleaning, and construction of the final dataset were completed between 3 June and 4 June 2026. Full bibliographic records and cited references were exported from the Web of Science Core Collection in BibTeX format and prepared for subsequent bibliometric analyses.

### 2.2. Inclusion and Exclusion Criteria and Data Extraction Process

Publications addressing artificial intelligence-based risk prediction, risk assessment, or risk stratification within anesthesiology, surgery, and the broader perioperative pathway were considered eligible for inclusion. Eligible publications examined preoperative, intraoperative, or postoperative risks and clinically relevant outcomes, including hemodynamic instability, mortality, postoperative complications, organ-specific morbidity, neurocognitive outcomes, intensive care unit admission, prolonged hospitalization, and other adverse perioperative outcomes. Clinical, methodological, conceptual, and preclinical studies were considered eligible when they contributed meaningfully to the development, evaluation, or application of AI-supported risk assessment, prediction, stratification, or clinical decision support within this broader context. This broad eligibility framework was adopted to capture not only predictive model-development and validation studies but also the clinical, methodological, and conceptual knowledge structure surrounding AI-based perioperative risk prediction.

The initial Web of Science Core Collection search identified 170 records. Following the sequential application of the predefined database-level restrictions, 18 records were excluded: six records indexed outside SCI-EXPANDED and ESCI, three German-language publications, and nine records classified as ineligible publication types. The latter group comprised editorial materials, meeting abstracts, and proceeding papers that did not meet the predefined document-type criteria. The thematic eligibility assessment required a meaningful clinical, methodological, or conceptual connection to AI-supported risk assessment, prediction, stratification, or decision support within anesthesiology, surgery, or the broader perioperative pathway. Thematically relevant preclinical studies were also considered eligible when they contributed to the understanding or development of AI-based perioperative risk prediction approaches.

**Figure 1:**
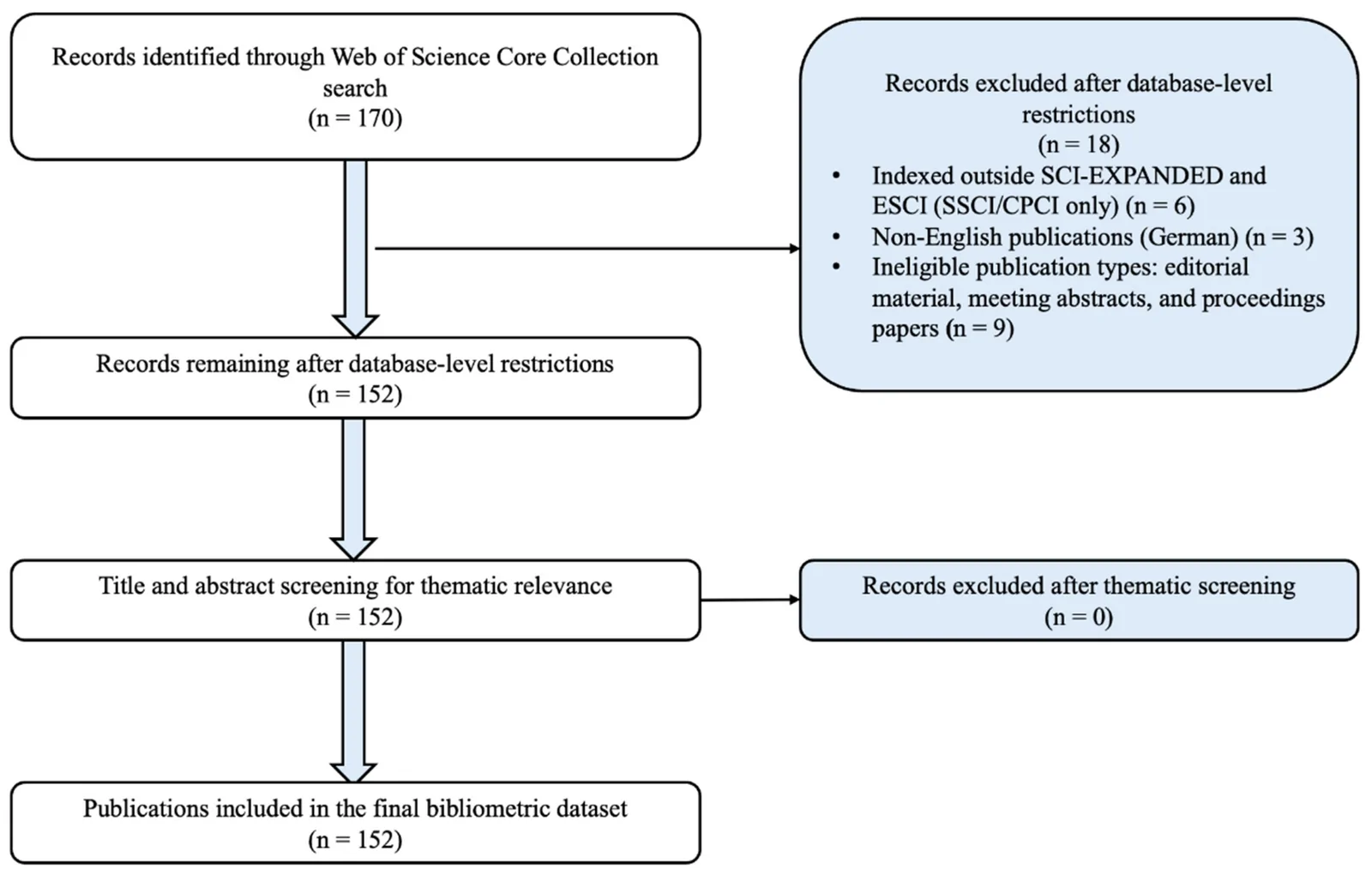
Flow diagram illustrating the identification, screening, filtering, and selection process of publications included in the bibliometric analysis.

In the original screening, the remaining 152 records were manually screened by the author on the basis of their titles and abstracts, with full-text review undertaken when thematic eligibility could not be determined reliably from the available bibliographic information. All records met the broadly defined thematic eligibility criteria, and no additional exclusions were made at this stage. This absence of thematic exclusions was likely related to the specificity of the three-block search strategy, which required the simultaneous presence of AI-, risk prediction-, and anesthesiology-related concepts. The record identification, database-level restriction, screening, eligibility assessment, and final selection process are presented in a PRISMA-style flow diagram in Figure 1.

During revision, the screening process was repeated using a broader set of AI-related search terms adapted from Xie et al. [24], while retaining the original risk prediction and anesthesiology concept blocks. The records retrieved through the expanded search were independently screened by the author and a second specialist in Anesthesiology and Reanimation according to the same predefined eligibility criteria, with disagreements resolved through discussion and consensus.

For each record, publication year, author information, institutional affiliations, country of origin, source journal, keywords, citation counts, and cited references were extracted. These data were subsequently used for analyses of scientific productivity, citation performance, collaboration networks, conceptual structure, and thematic development. This study was not registered in a public review registry.

### 2.3. Bibliometric Analysis and Science Mapping

Bibliometric analyses were performed using the Bibliometrix package (version 5.4.0) and the Biblioshiny web interface (version 5.0) within the R statistical environment (R Foundation for Statistical Computing, Vienna, Austria; version 4.5.2) [28].

The complete analytical settings used for the network, co-citation, keyword co-occurrence, thematic map, and thematic evolution analyses are provided in Supplementary Table S1.

Descriptive bibliometric indicators were first evaluated. Annual scientific production trends, the most productive authors, institutions, countries, and journals were identified. The compound annual growth rate (CAGR) was calculated using complete publication years according to the formula CAGR = $[(N_{f}/N_{i}{)}^{1/t}-1]\times 100$, where $N_{i}$ and $N_{f}$ represent the publication counts in the first and last complete years, respectively, and t represents the number of elapsed annual intervals. The partial 2026 data were excluded from this calculation. Citation performance was also assessed to determine the most influential publications and the studies forming the intellectual foundation of the field.

To characterize the structure of scientific collaboration, international collaboration networks were analyzed. Country-level scientific production was calculated using distinct document counts based on author-affiliation addresses. Each country was counted only once per publication, irrespective of the number of authors or institutional affiliations from that country. In internationally co-authored publications, each contributing country received one document count. In network visualizations, nodes represented authors, institutions, countries, or keywords, whereas edges represented the relationships among these entities. Node size reflected bibliometric weight, while edge thickness indicated the strength of the association.

To explore the conceptual structure of the field, co-occurrence analysis was performed using author keywords. Keyword networks were used to identify major themes, conceptual clusters, and research foci. In addition, trend topic analysis was conducted to examine changes in research interests over time.

Thematic map and thematic evolution analyses were performed to investigate the knowledge structure and thematic development of the research field. In the thematic map, themes were classified according to their centrality and density values as motor themes, basic themes, niche themes, and emerging or declining themes. Thematic evolution analysis was used to examine the temporal development of research themes and their interrelationships.

Finally, citation analyses were conducted to identify the knowledge base and influential contributions within the field. Global citation metrics, highly cited publications, and major research contributions were evaluated. The findings were presented through tables, network maps, and bibliometric visualizations.

## 3. Results

### 3.1. Dataset Characteristics and Temporal Growth Trends

A total of 152 publications indexed in the WoS were included in the analysis. The publications were distributed across 98 sources and produced by 986 authors between 2018 and 2026. Based on complete publication years, the compound annual growth rate of the literature between 2018 and 2025 was 77.72%; the partial 2026 data were excluded from this calculation. The collaboration index was 6.95, and the international co-authorship rate was 17.11%. Original research articles accounted for the majority of publications (*n* = 116, 76.3%), followed by review articles (*n* = 34, 22.4%) and early access publications (*n* = 2, 1.3%) (Table 1).

**Figure 2:**
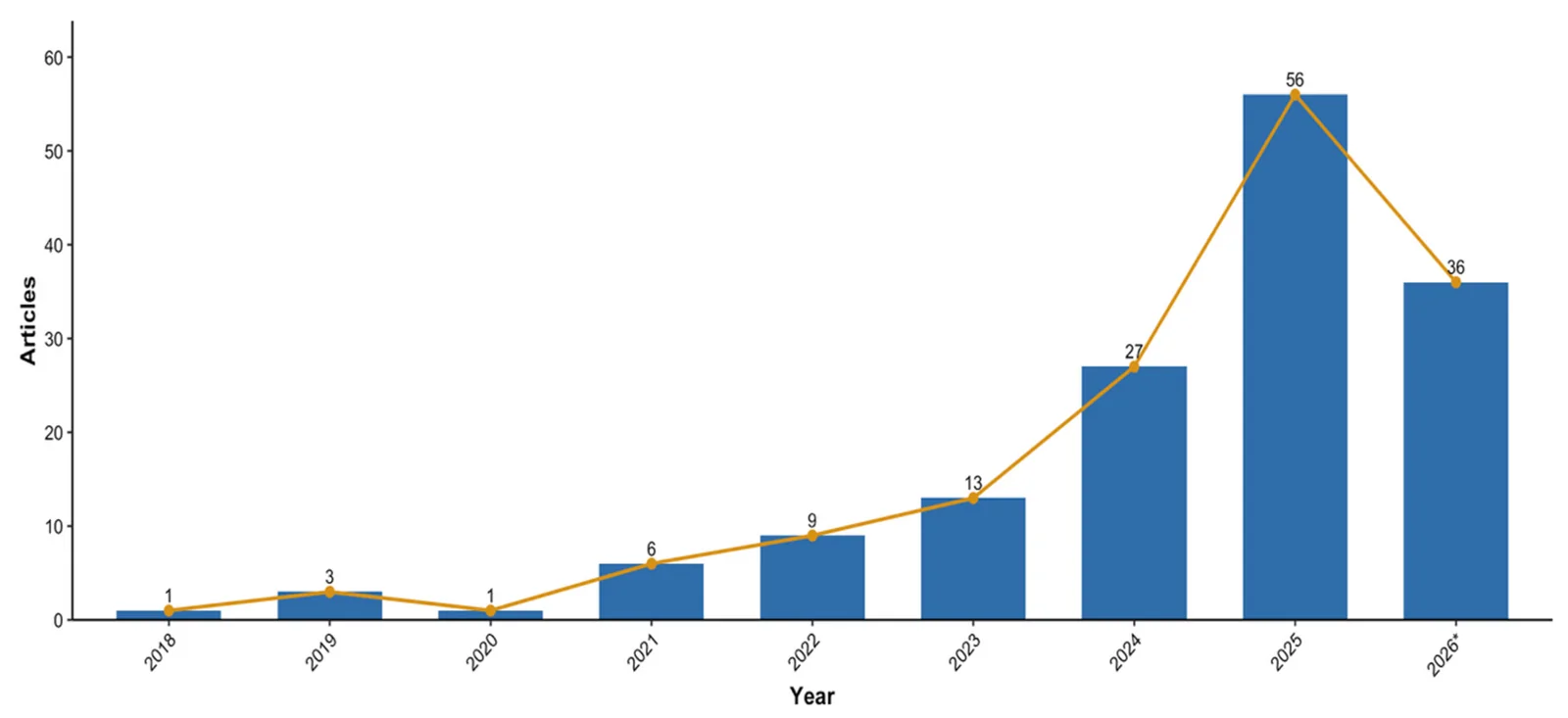
Annual scientific production (2018–3 June 2026): * 2026 represents a partial year and was excluded from the growth-rate calculation.

Scientific production increased substantially across the complete publication years analyzed (Figure 2). Following a limited publication volume between 2018 and 2020, annual output increased progressively from 2021 onward. Publication counts rose from 6 in 2021 to 13 in 2023, followed by a marked increase to 27 publications in 2024 and 56 publications in 2025, representing the highest output among the complete publication years. A total of 36 publications were indexed between 1 January and 3 June 2026. Because the 2026 value represents a partial year, it was not interpreted as an annual decline and was excluded from the compound annual growth-rate calculation.

### 3.2. Journal Analysis and Bradford Core

The 152 publications were distributed across 98 journals. Current Opinion in Anesthesiology was the most productive source (*n =* 8), followed by British Journal of Anaesthesia (*n =* 7), BMJ Open (*n* = 6), Journal of Clinical Medicine (*n* = 6), and BMC Medical Informatics and Decision Making (*n* = 5). Additional contributions were identified in Frontiers in Medicine, Journal of Clinical Anesthesia, Journal of Medical Internet Research, and PLOS ONE (Table 2).

Bradford’s Law analysis demonstrated a concentration of publications within a limited number of core journals. The Bradford core consisted of Current Opinion in Anesthesiology, British Journal of Anaesthesia, BMJ Open, Journal of Clinical Medicine, and BMC Medical Informatics and Decision Making. These journals collectively represented the principal publication outlets for research on artificial intelligence-based perioperative risk prediction.

### 3.3. Authors, Institutions, and Scientific Collaboration

Author productivity analysis revealed a highly distributed authorship structure. No individual author contributed more than three publications, indicating the absence of a single dominant research group within the field. This pattern suggests that scientific production has been generated by multiple independent research teams rather than concentrated around a small number of investigators.

Institutional productivity was led by Seoul National University and Yonsei University (17 publications each), followed by Washington University (*n* = 16), Heidelberg University (*n* = 11), and the University of Parma (*n* = 11). Other major contributors included the Catholic University of Korea, the University of California Los Angeles, Albert Einstein College of Medicine, Emory University, the University of California San Francisco, and the University of Washington. Country-level analysis identified the United States as the leading contributor (*n* = 50), followed by China (*n* = 48), Italy (*n* = 11), Germany (*n* = 10), and South Korea (*n* = 8). The most productive countries and institutions are summarized in Table 3.

**Figure 3:**
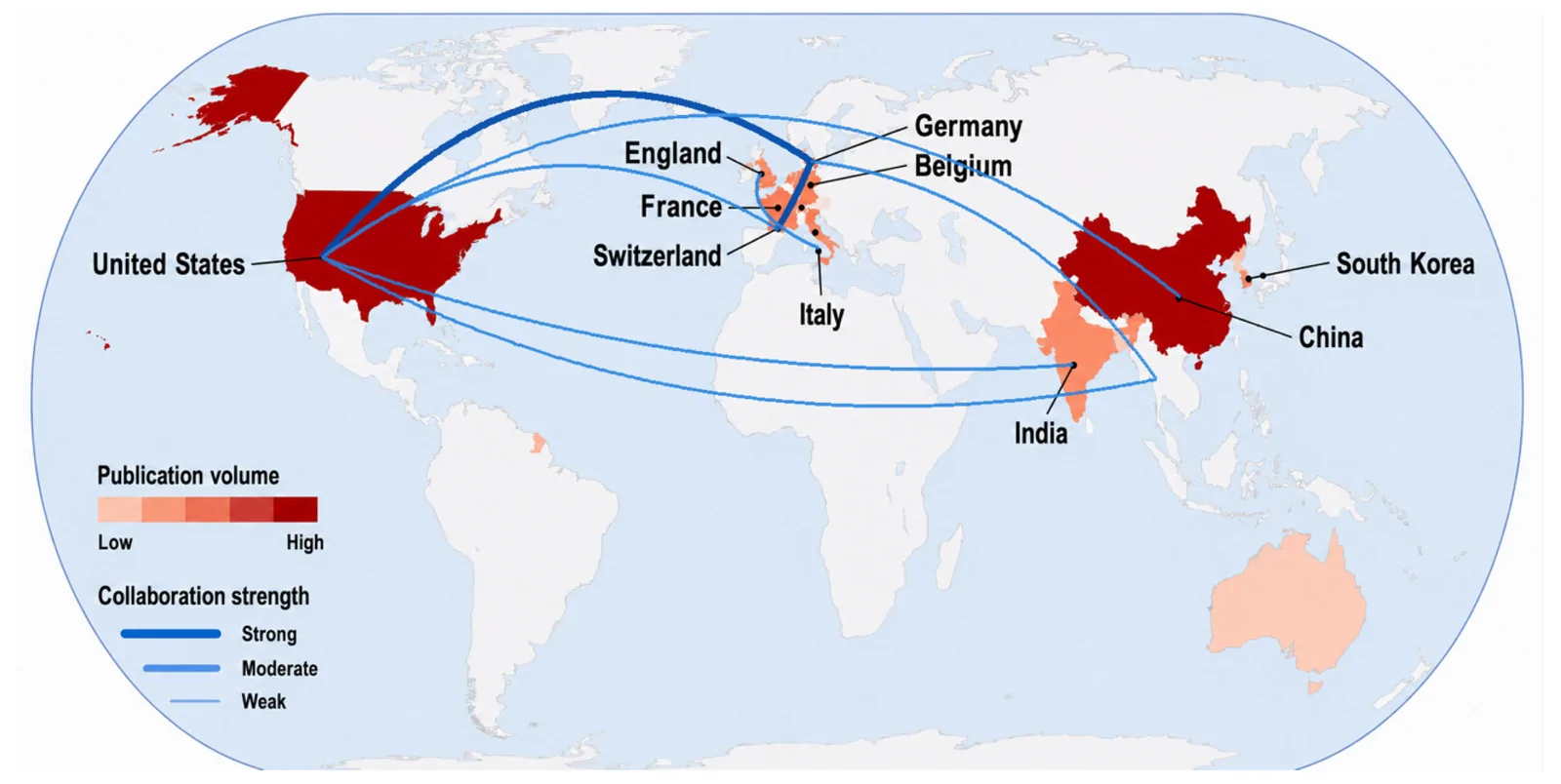
Country Collaboration Map of AI-Based Perioperative Risk Prediction Research.

International collaboration was concentrated within a limited number of country pairs (Figure 3). After applying a minimum threshold of two joint publications, eight collaborative links were identified. The strongest collaborations were observed between Germany and Switzerland and between the United States and Germany (*n* = 3 each). China–United States, Germany–Thailand, Italy–United Kingdom, United States–India, United States–Switzerland, and United States–Thailand each accounted for two joint publications.

### 3.4. Citation Metrics and Intellectual Structure

At the document level, Hashimoto et al. (2020) [9] was the most highly cited publication in the dataset, with 354 global citations. Other highly cited studies included Kendale et al. (2018) [12] and Fritz et al. (2019) [4], with 178 and 92 global citations, respectively. Chung et al. (2024) [29] and Bishara et al. (2022) [30] also ranked among the five most highly cited publications. The publications with the highest global citation counts are presented in Table 4.

Local citation analysis indicated that the intellectual structure of the literature was primarily organized around the works of Hashimoto et al. (2020) [9], Hatib et al. (2018) [33], Kendale et al. (2018) [12], Lundberg et al. (2018) [34], and Wijnberge et al. (2020) [35]. These references represented important methodological and clinical foundations of the field.

**Figure 4:**
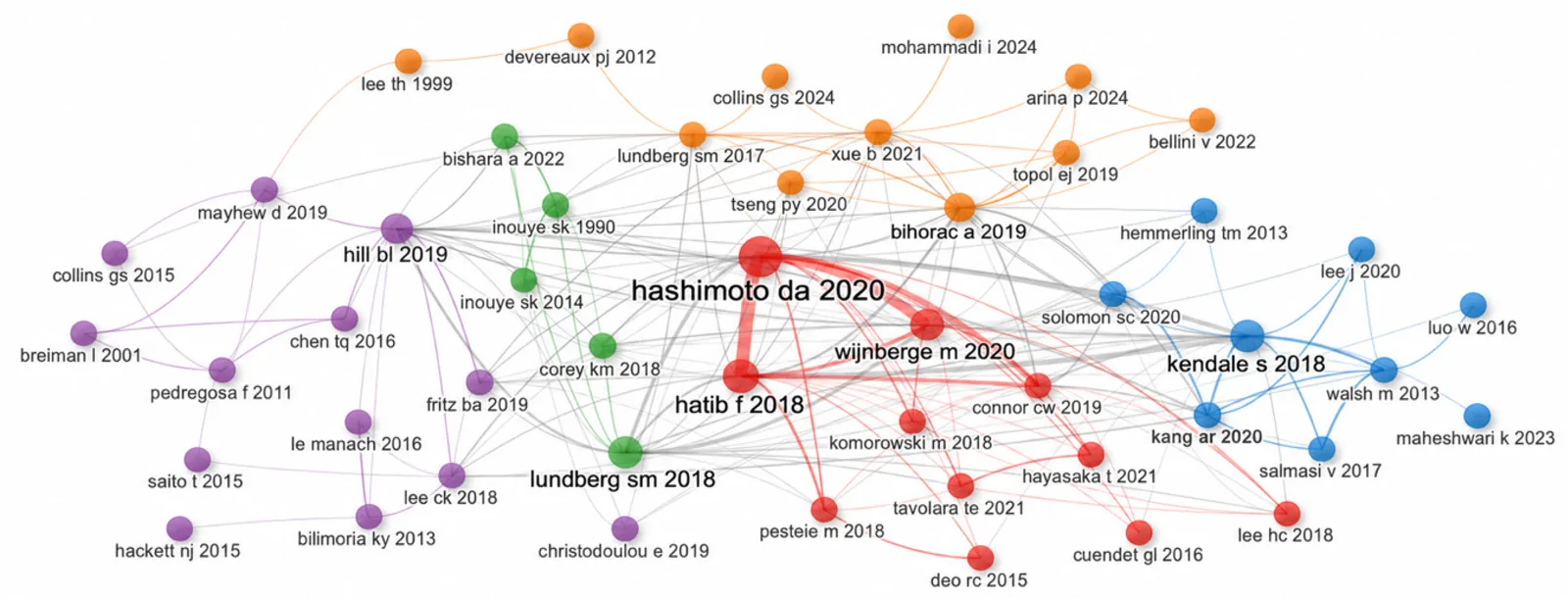
Reference co-citation network illustrating the intellectual structure of AI-based perioperative risk prediction research. Colors represent distinct co-citation clusters identified within the network.

Reference co-citation analysis further confirmed the central roles of Hashimoto et al. (2020) [9], Kendale et al. (2018) [12], Hatib et al. (2018) [33], Lundberg et al. (2018) [34], and Hill et al. (2019) [8] within the intellectual structure of AI-based perioperative risk prediction research (Figure 4).

**Figure 5:**
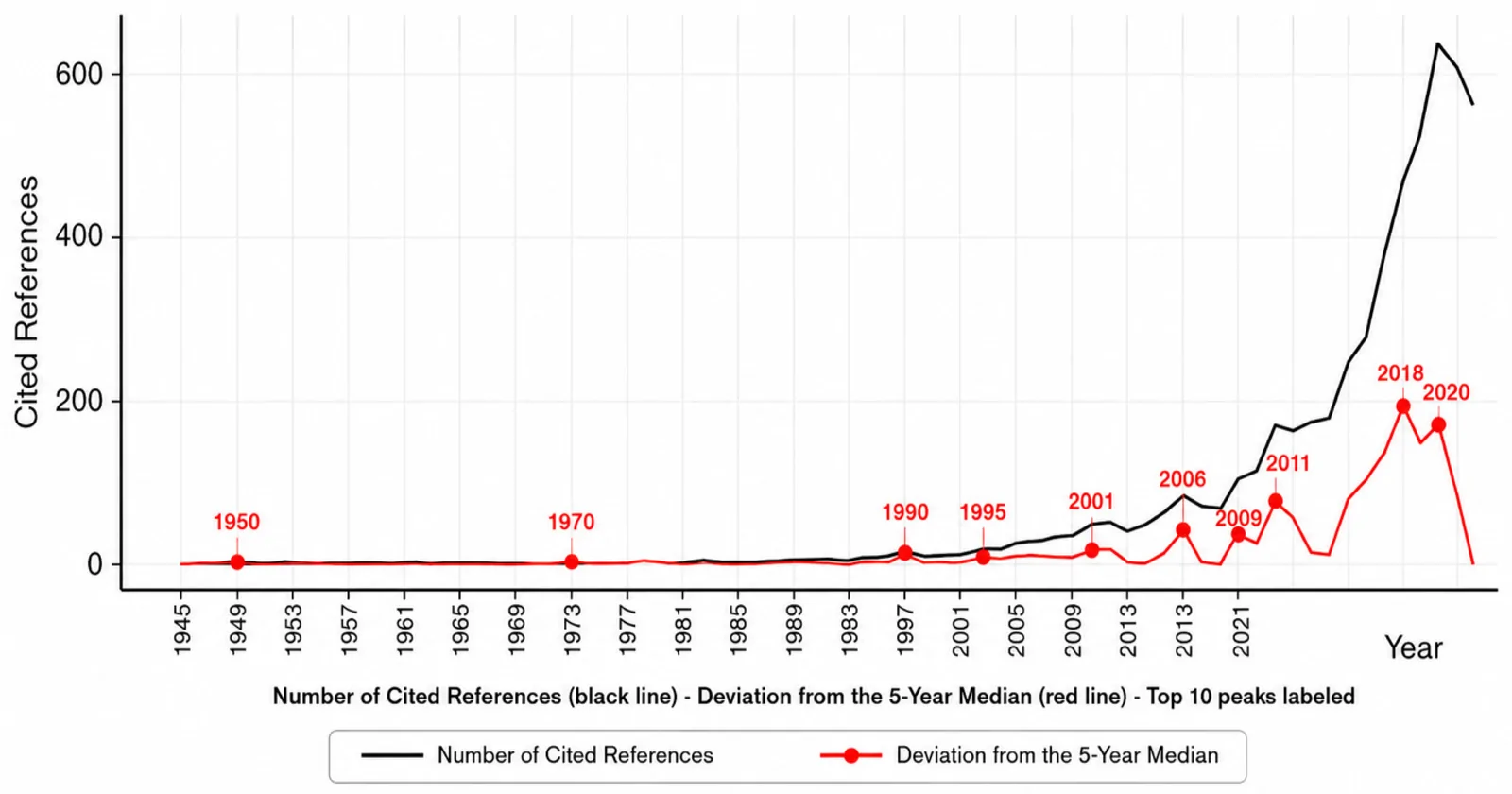
Reference Publication Year Spectroscopy (RPYS) of AI-based perioperative risk prediction research.

Reference Publication Year Spectroscopy (RPYS) identified several prominent citation peaks across the historical development of the field. The most pronounced peaks occurred in 2018 and 2020, corresponding to influential publications on machine learning-based perioperative prediction, predictive hemodynamic monitoring, and artificial intelligence applications in anesthesiology. Earlier peaks observed in 2001, 2006, 2009, and 2011 reflected foundational contributions related to risk modeling, statistical prediction methods, and clinical decision-support systems (Figure 5).

### 3.5. Conceptual Structure and Research Trends

Keyword analysis identified “machine learning” (frequency = 74) and “artificial intelligence” (frequency = 46) as the dominant concepts within the literature, followed by “anesthesia” (frequency = 33), “surgery” (frequency = 32), and “outcomes” (frequency = 20). Additional frequently occurring terms included “prediction,” “risk,” “mortality,” “complications,” “management,” and “risk stratification.”

Trend topic analysis demonstrated a substantial increase in the occurrence of machine learning- and artificial intelligence-related terms after 2022. Simultaneously, clinically oriented concepts such as mortality, complications, outcomes, and risk stratification became increasingly prominent, indicating a growing focus on perioperative outcome prediction and clinical risk assessment.

**Figure 6:**
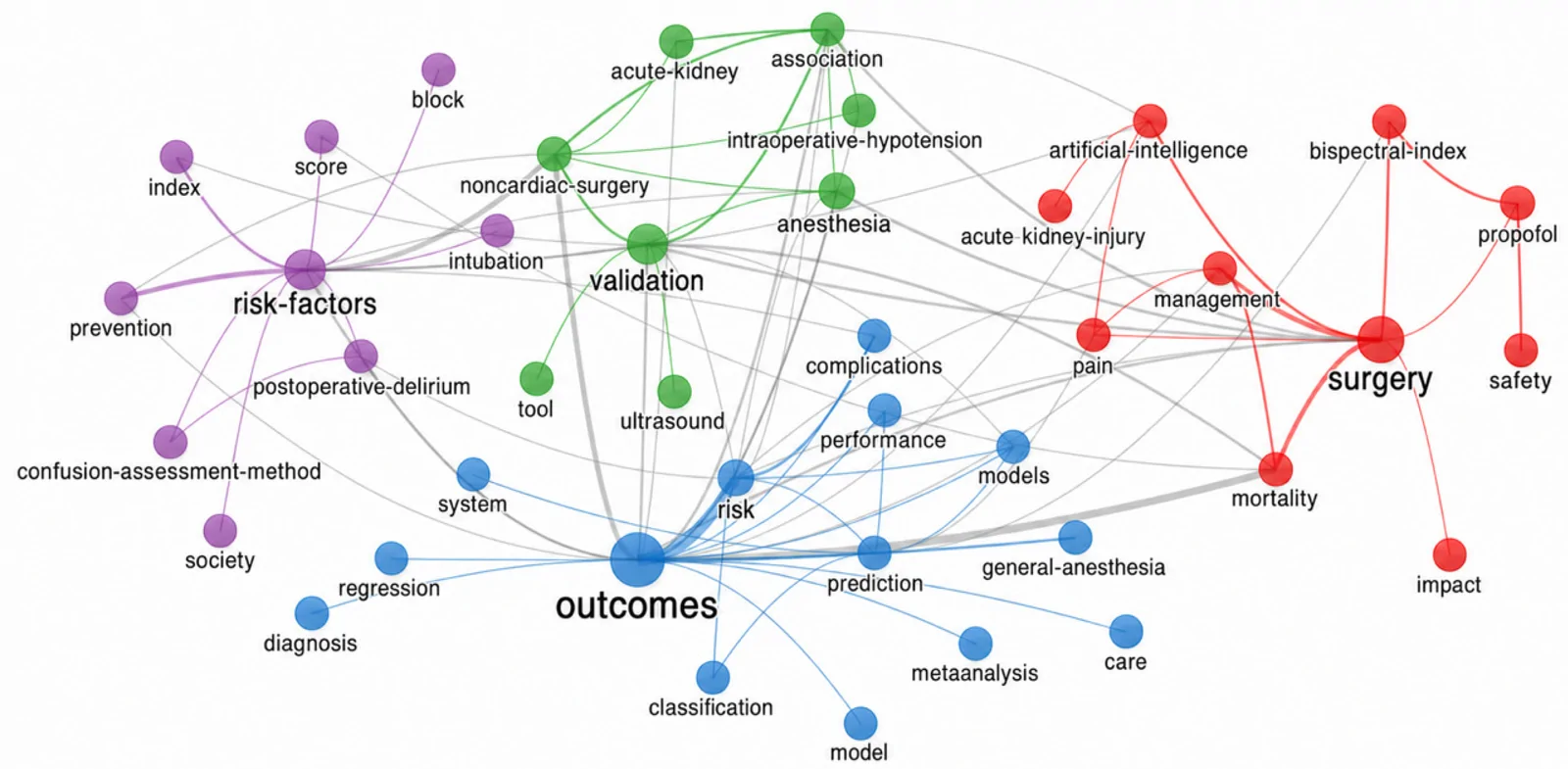
Author–keyword co-occurrence network illustrating the conceptual structure of AI-based perioperative risk prediction research. Colors represent distinct keyword clusters identified by the co-occurrence analysis.

Author–keyword co-occurrence analysis identified four interconnected conceptual clusters (Figure 6). The first cluster centered on risk factors, prevention, and postoperative delirium; the second linked anesthesia and validation with intraoperative hypotension, noncardiac surgery, and ultrasound; the third was organized around outcomes, risk, prediction, complications, and model performance; and the fourth connected surgery and artificial intelligence with mortality, pain, management, and safety. Collectively, these clusters demonstrate the close relationship between methodological development, perioperative risk assessment, and clinically relevant outcomes.

### 3.6. Thematic Structure and Evolution

The thematic map revealed that “machine learning” and “prediction model” constituted the principal motor themes of the field, characterized by both high centrality and high density. In contrast, “artificial intelligence,” “anesthesiology,” “perioperative care,” and “risk prediction” were positioned as basic themes, indicating their central role within the conceptual framework of the literature.

**Figure 7:**
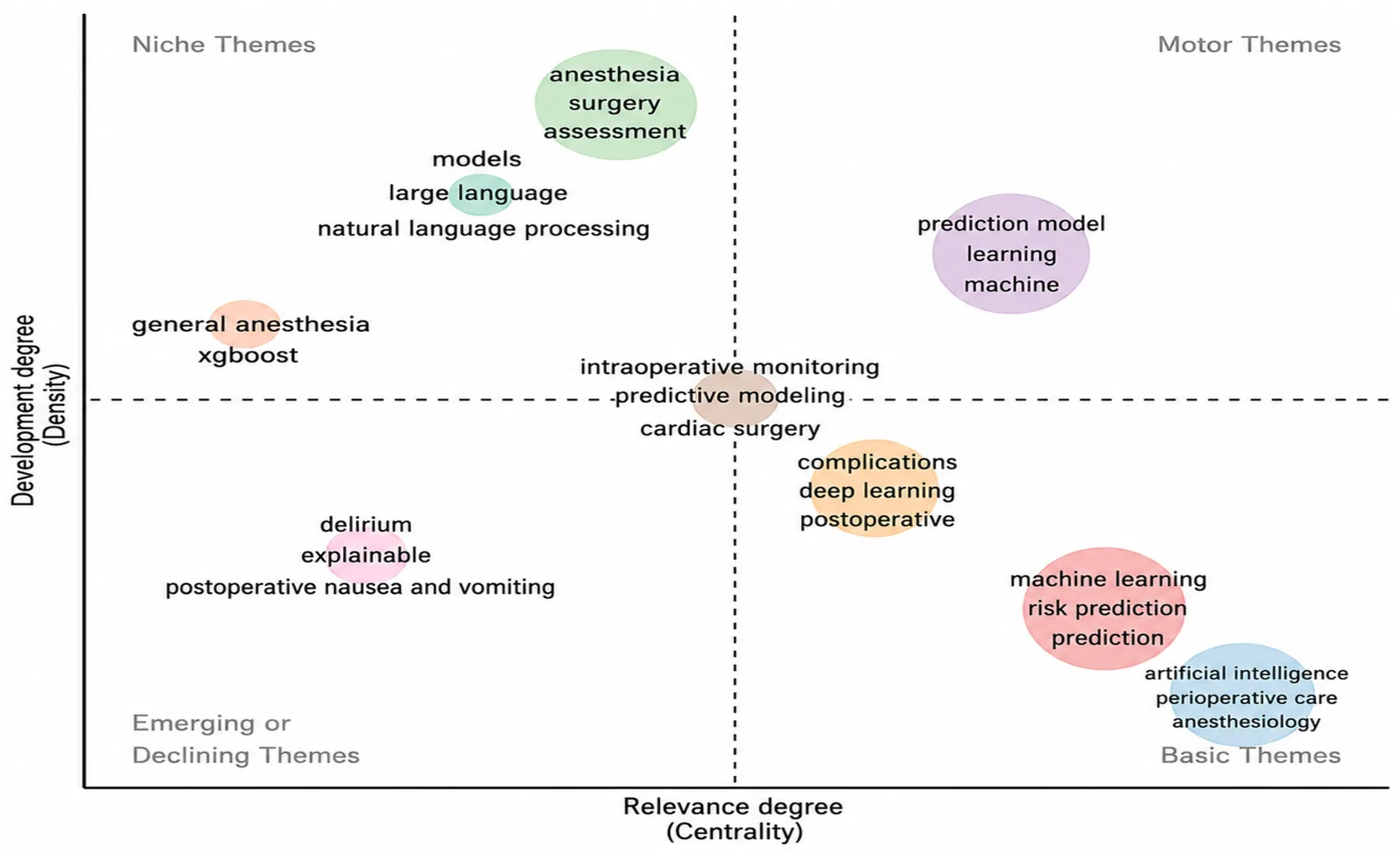
Thematic map showing motor, basic, niche, and emerging themes according to centrality and density measures.

Emerging themes included “explainable,” “delirium,” and “postoperative nausea and vomiting,” whereas “large language models,” “natural language processing,” and “assessment” appeared as niche themes. These findings indicate the presence of both clinically oriented and methodologically innovative research directions within the field (Figure 7).

**Figure 8:**
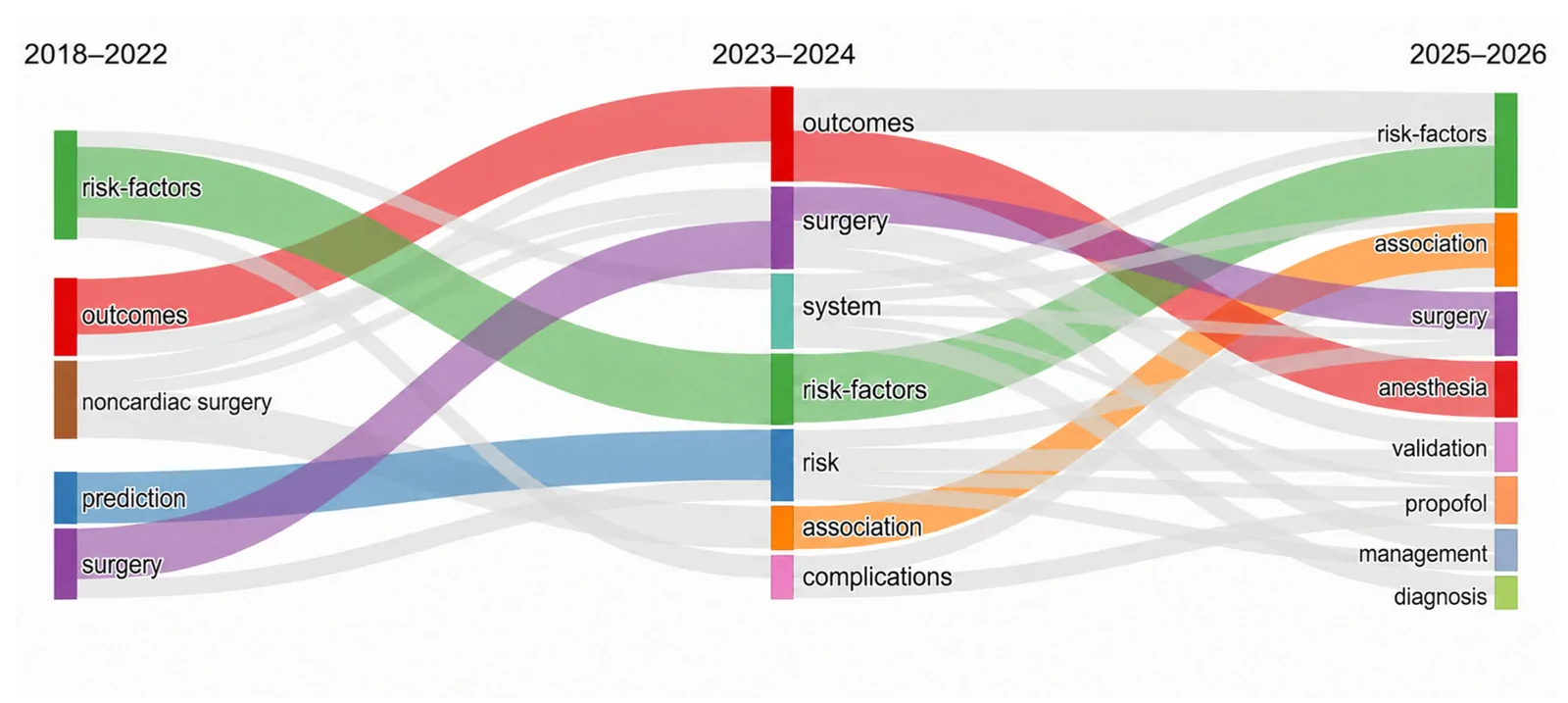
Thematic evolution of AI-based perioperative risk prediction research across the periods 2018–2022, 2023–2024, and 2025–2026.

Thematic evolution analysis demonstrated a progressive shift in research focus over time. Early studies (2018–2022) primarily concentrated on artificial intelligence and machine learning applications in anesthesia. During 2023–2024, themes related to risk stratification, complications, and perioperative outcomes became increasingly prominent. In the most recent period (2025–2026), prediction models, predictive analytics, and machine learning emerged as the dominant thematic clusters, reflecting the growing emphasis on clinically applicable risk prediction systems in anesthesiology (Figure 8).

## 4. Discussion

### 4.1. Main Findings

This bibliometric analysis provides a structured overview of artificial intelligence (AI)-based risk prediction research in anesthesiology. Four principal findings emerged. First, scientific production increased markedly after 2023, indicating growing interest in perioperative AI applications. Second, the intellectual foundations of the field were largely shaped by influential studies published between 2018 and 2020, particularly those addressing machine learning-based prediction, intraoperative hypotension forecasting, and AI applications in anesthesiology. Third, the conceptual structure of the literature showed a transition from general AI and machine learning research toward clinically oriented risk stratification, complication prediction, and decision-support systems. Finally, emerging themes such as explainable AI, natural language processing, and large language models suggest that the field is moving toward more interpretable and clinically integrated prediction systems.

### 4.2. Rapid Growth of Ai-Based Risk Prediction Research

The rapid increase in publication output after 2023 suggests that AI-based risk prediction has moved from an exploratory research area toward a more visible domain within perioperative medicine. This growth is likely related to the increasing availability of electronic health records, high-frequency intraoperative monitoring data, and large perioperative databases. These data sources have created opportunities for models that can integrate demographic, laboratory, comorbidity, surgical, anesthetic, and physiological variables into individualized risk estimates [1,5,10].

From a clinical perspective, this growth aligns with a broader shift in perioperative medicine from reactive management toward anticipatory care. Anesthesiology is a data-rich specialty in which hemodynamic variables, ventilatory parameters, drug administration patterns, laboratory results, and postoperative outcomes are continuously generated. Therefore, the specialty provides a favorable environment for predictive modeling. However, the expansion of AI research does not necessarily imply immediate clinical adoption. Translation into practice requires external validation, workflow integration, prospective evaluation, and demonstration that model-guided interventions improve clinically meaningful outcomes rather than only statistical performance [2,17].

The multidisciplinary distribution of publications across anesthesiology, perioperative medicine, biomedical informatics, and digital health journals reflects this translational challenge. AI-based perioperative risk prediction is not only a computational problem but also a clinical implementation problem involving anesthesiologists, surgeons, intensivists, informaticians, and health system stakeholders [36,37,38].

### 4.3. Intellectual Foundations of the Field

Citation and co-citation analyses showed that the field is strongly influenced by a limited number of foundational studies. Hashimoto et al. [9], Kendale et al. [12], Hatib et al. [33], Hill et al. [8], Fritz et al. [4], Lundberg et al. [34], and Wijnberge et al. [35] occupied central positions within the citation structure. These publications are not merely bibliometric landmarks; they also represent key clinical and methodological turning points in the development of AI-assisted perioperative care.

Hashimoto et al. provided one of the most influential syntheses of AI applications in anesthesiology and organized the field around clinically relevant domains, including depth-of-anesthesia monitoring, anesthetic control, event and risk prediction, ultrasound guidance, pain management, and operating room logistics [9]. This framework helped position risk prediction as one of the core clinical applications of AI in anesthesiology rather than as a purely computational exercise.

The prominence of studies on intraoperative hypotension prediction reflects the central role of this field in the clinical translation of AI-based monitoring. Hypotension is clinically important because it has been associated with myocardial injury, acute kidney injury, and mortality [35]. Machine learning-derived early warning systems such as the Hypotension Prediction Index (HPI) have attempted to move prediction from retrospective risk modeling toward real-time preventive hemodynamic management [33]. The HYPE randomized trial suggested that an HPI-guided protocol could reduce the depth and duration of intraoperative hypotension compared with standard care [35], although questions regarding generalizability, outcome relevance, and implementation requirements remain.

Fritz et al. (2019) [4] and Kendale et al. (2018) [12] represent another branch of the intellectual foundation: perioperative prediction based on large-scale clinical data. These studies demonstrated the feasibility of applying machine learning to predict perioperative outcomes and helped shift attention from single-parameter monitoring toward multivariable risk prediction models [4,12]. Lundberg et al. (2020) introduced the SHAP framework, which became influential because it addressed one of the major barriers to clinical adoption: explaining why a model generates a specific prediction [34].

Taken together, these foundational studies show that the field has developed through two interacting streams: clinically driven prediction problems, such as hypotension and postoperative complications, and methodological advances, such as explainability and model interpretation.

### 4.4. Transition from Algorithm Development to Clinical Risk Prediction

The conceptual and thematic analyses indicated a shift from general AI methodology toward clinically specific risk prediction. Early studies largely focused on feasibility, model performance, and algorithmic development. The more recent literature increasingly emphasizes perioperative outcomes such as mortality, complications, delirium, postoperative nausea and vomiting, and broader risk stratification.

This transition is important because perioperative risk prediction has limited value if it remains detached from clinical decision-making. For anesthesiologists, a prediction model is clinically meaningful only when it can support an actionable intervention. In elective surgery, AI-assisted preoperative risk stratification may help identify patients at increased risk of postoperative pulmonary complications and inform individualized respiratory optimization, anesthetic and ventilatory planning, and enhanced postoperative surveillance [1,5,6,7]. Predictive models may also contribute to complication prevention by supporting earlier hemodynamic treatment, postoperative triage, and targeted monitoring. Therefore, the growing prominence of “risk stratification,” “complications,” “mortality,” and “outcomes” in the conceptual structure suggests a gradual movement toward clinically actionable endpoints [1,39].

Postoperative delirium emerging as a thematic signal is consistent with increasing interest in geriatric perioperative care and neurocognitive outcomes [30,40]. Delirium is multifactorial, time-sensitive, and dependent on patient vulnerability, anesthetic exposure, inflammation, pain, sleep disruption, and postoperative complications [40]. These characteristics make it a suitable but challenging target for AI-based prediction. Similarly, postoperative nausea and vomiting represent a patient-centered recovery outcome in which risk prediction may support prophylaxis selection and individualized perioperative planning [16,41].

The relatively less distinct clustering of acute kidney injury (AKI) requires careful interpretation. Clinically, AKI is one of the most important postoperative complications and has been a frequent target of machine learning models [42,43]. However, in this bibliometric analysis, AKI did not emerge as an independent dominant thematic cluster. This may be because AKI is often modeled as part of broader postoperative morbidity, organ dysfunction, critical care, hemodynamic instability, or mortality prediction frameworks rather than appearing as a separate keyword cluster. It may also reflect variability in author keyword selection, where studies addressing renal outcomes may use broader terms such as “complications,” “critical care,” “outcomes,” or “organ dysfunction.” Therefore, the absence of AKI as an independent cluster should not be interpreted as indicating low clinical relevance; rather, it suggests that AKI-related prediction research may be embedded within broader perioperative complication and mortality modeling.

### 4.5. Emerging Themes and Future Directions

Explainable artificial intelligence (XAI) emerged as an important developing theme. This finding suggests a growing emphasis on transparency and interpretability in perioperative AI research. In anesthesiology, explainability is not only a technical preference but also a clinical and medicolegal requirement, as prediction models increasingly influence perioperative decision-making. Consequently, successful implementation of AI systems will depend not only on predictive accuracy but also on clinicians’ ability to understand and trust model outputs [17,34].

Explainability is particularly relevant in perioperative medicine because similar risk estimates may arise from different clinical profiles. For example, a predicted risk of intraoperative hypotension may reflect baseline hemodynamic status, anesthetic exposure, comorbidity burden, or recent physiological instability. Interpretability therefore helps clinicians determine whether a prediction is clinically plausible and actionable [34].

Large language models and natural language processing appeared as niche themes. Their current position suggests that they are not yet fully integrated into mainstream perioperative risk prediction research, but their emergence is notable. Much perioperative information is stored in unstructured text, including preoperative assessments, anesthesia records, operative notes, ICU notes, and discharge summaries. NLP and large language models may support the extraction of clinically relevant information from these sources and help integrate structured and unstructured data into future prediction systems [22]. However, evidence directly comparing chatbot-based perioperative risk stratification with conventional clinical judgment remains limited and is of very low certainty [44]. Prospective studies should evaluate predictive discrimination, calibration, consistency, safety, and incremental clinical utility before these tools are considered for autonomous or broader perioperative risk assessment.

Digital twins and multimodal prediction systems may represent longer-term directions [45]. Perioperative risk is dynamic rather than static; it evolves from preoperative evaluation through intraoperative management and postoperative recovery. Future AI systems may therefore need to combine preoperative risk factors, intraoperative physiological streams, medication exposure, surgical events, and postoperative trajectories. Recent specialty-specific applications include deep learning-based prediction of post-hepatectomy liver failure in hepatocellular carcinoma and AI-supported risk stratification, surgical planning, and postoperative outcome prediction in oncologic thoracic surgery [46,47]. However, the clinical value of such systems will depend on prospective validation, transparent reporting, integration into workflow, and evidence that model-guided care improves outcomes.

### 4.6. Strengths and Limitations

This study has several strengths. By integrating productivity indicators, citation analyses, collaboration networks, RPYS, thematic mapping, and thematic evolution, it provides a comprehensive overview of the scientific and conceptual development of AI-based perioperative risk prediction research.

Several limitations should be acknowledged. First, the analysis was restricted to the WoS and may not have captured all relevant publications indexed in other databases. In addition, the focused Topic-field search strategy required the simultaneous presence of artificial intelligence-, risk-, and anesthesiology-related terms and may therefore have excluded influential publications framed more broadly within the field. Second, citation-based metrics are influenced by publication age and citation practices, potentially underestimating the impact of recent studies. Third, thematic analyses depend on author-selected keywords and database metadata, which may affect the representation of specific topics. Finally, bibliometric methods describe research trends and knowledge structures but cannot evaluate the clinical effectiveness of AI systems.

Despite these limitations, this study offers a structured overview of the evolution of AI-based perioperative risk prediction and highlights emerging directions for future research.

## 5. Conclusions

This bibliometric analysis provides an overview of the scientific and conceptual development of artificial intelligence-based risk prediction research in anesthesiology. The findings suggest an increasing emphasis on clinically relevant outcomes and perioperative decision support, while also highlighting emerging areas that may shape future research in the field.

## Figures and Tables

**Table 1:** Main characteristics of the bibliometric dataset.

Characteristic	Value
Timespan	2018–3 June 2026
Documents	152
Source journals	98
Authors	986
Authors of single-authored documents	2
Single-authored documents	2
Co-authors per document	6.95
International co-authorships (%)	17.11
Keywords Plus (ID)	385
Author Keywords (DE)	476
Unique cited references	5704
Mean cited references per document	44.61
Compound annual growth rate (%)	77.72 *
Document age, median (IQR), years	1.0 (1.0–2.0)
Average citations per document	10.84
Articles	116
Articles (early access)	2
Reviews	34

* The compound annual growth rate was calculated using complete publication years from 2018 to 2025. The partial 2026 data were excluded from this calculation.

**Table 2:** Most productive sources contributing to AI-based perioperative risk prediction research.

Rank	Source	Publications
1	Current Opinion in Anesthesiology	8
2	British Journal of Anaesthesia	7
3	BMJ Open	6
3	Journal of Clinical Medicine	6
5	BMC Medical Informatics and Decision Making	5
6	Frontiers in Medicine	4
6	Journal of Clinical Anesthesia	4
6	Journal of Medical Internet Research	4
6	PLOS ONE	4
10	BMC Anesthesiology	3

**Table 3:** Most productive countries and institutions contributing to AI-based perioperative risk prediction research.

**Panel A. Most Productive Countries by Number of Unique Publications**		
**Rank**	**Country**	**Publications, *n***
1	United States	50
2	China	48
3	Italy	11
4	Germany	10
5	South Korea	8
6	England	7
6	France	7
8	India	6
9	Switzerland	5
10	Belgium	4
**Panel B. Most productive institutions**		
**Rank**	**Institution**	**Publications**
1	Seoul National University	17
1	Yonsei University	17
3	Washington University	16
4	Heidelberg University	11
4	University of Parma	11
6	Catholic University of Korea	10
6	University of California, Los Angeles	10
8	Albert Einstein College of Medicine	9
8	Emory University	9
8	University of California, San Francisco	9
8	University of Washington	9

Note: Values represent distinct document counts. Each country was counted once per publication; internationally co-authored publications were assigned once to each contributing country.

**Table 4:** Most influential publications within the final bibliometric dataset according to global citation counts.

Rank	Publication	Journal	Global Citations, *n*
1	Hashimoto et al. (2020) [9]	Anesthesiology	354
2	Kendale et al. (2018) [12]	Anesthesiology	178
3	Fritz et al. (2019) [4]	British Journal of Anaesthesia	92
4	Chung et al. (2024) [29]	JAMA Surgery	78
5	Bishara et al. (2022) [30]	BMC Anesthesiology	74
6	Bellini et al. (2022) [1]	Journal of Anesthesia, Analgesia and Critical Care	63
7	Hill et al. (2019) [8]	British Journal of Anaesthesia	62
8	Bellini et al. (2022) [10]	Minerva Anestesiologica	39
9	Ding et al. (2025) [31]	Journal of Medical Internet Research	38
10	Wongtangman et al. (2023) [32]	Journal of Clinical Anesthesia	32

## Data Availability

The bibliographic data analyzed during the current study were retrieved from the Web of Science Core Collection database. The dataset and analysis files are available from the corresponding author upon reasonable request.

## Supplementary Materials

The following supporting information can be downloaded at: https://www.mdpi.com/article/10.3390/healthcare14172785/s1, Table S1. Analytical parameters used for network and thematic analyses.

## Abbreviations

AI	Artificial Intelligence
AKI	Acute Kidney Injury
CAGR	Compound Annual Growth Rate
DL	Deep Learning
ESCI	Emerging Sources Citation Index
HPI	Hypotension Prediction Index
ICU	Intensive Care Unit
LLM	Large Language Model
ML	Machine Learning
NLP	Natural Language Processing
PONV	Postoperative Nausea and Vomiting
PRISMA	Preferred Reporting Items for Systematic Reviews and Meta-Analyses
RPYS	Reference Publication Year Spectroscopy
SCI-EXPANDED	Science Citation Index—Expanded
SHAP	SHapley Additive exPlanations
WoS	Web of Science
XAI	Explainable Artificial Intelligence
