# Supplementary material for: Artificial Intelligence for Perioperative Risk Prediction in Anesthesiology: A Bibliometric and Knowledge-Mapping Analysis
Source: Healthcare (Basel). 2026 Sep 1;14(17):2785. doi: 10.3390/healthcare14172785 (PMC13565178; doi:10.3390/healthcare14172785)
Supplement: Supplementary file 1 [file healthcare-14-02785-s001.zip › healthcare-4401947-supplementary.pdf]

Supplementary Table S1. Analytical parameters used for network and thematic analyses

Figure	Analysis and data field	Counting method	Selection criteria, thresholds, and displayed items	Normalization or weighting	Clustering and layout
Figure 3	Country collaboration map; countries derived from author-affiliation addresses	Full counting with within-document deduplication; each country was counted once per publication	Minimum collaboration frequency = 2 joint publications; 8 country pairs and 8 links displayed	None	Not applicable
Figure 4	Reference co-citation network; cited references (CR)	Full counting	50 references initially selected; isolated nodes removed; minimum number of edges = 2; 47 connected reference nodes displayed; no separate citation threshold	None additionally selected	Louvain clustering; automatic layout; repulsion force = 0.5
Figure 6	Author-keyword co-occurrence network; Author Keywords (DE)	Full counting	50 keywords initially selected; isolated nodes removed; minimum number of edges = 2; 41 connected keyword nodes displayed; no separate occurrence threshold	Association normalization	Louvain clustering; automatic layout; repulsion force = 0.5
Figure 7	Thematic map; Author Keywords (DE)	Full counting	Maximum number of words = 250; minimum cluster frequency = 5 per 1,000 documents; 9 thematic clusters displayed	Callon centrality and density; α = 0.5	Louvain clustering; community repulsion = 0.5
Figure 8	Thematic evolution; Author Keywords (DE)	Full counting	Maximum number of words = 250; minimum cluster frequency = 5 per 1,000 documents; cutting years = 2022 and 2024, producing three periods: 2018–2022, 2023–2024, and 2025–2026	α = 0.5; minimum weight index = 0.1	Louvain clustering
